# The Migrant-Local Difference in the Relationship Between Social Support, Sleep Disturbance, and Loneliness Among Older Adults in China: Cross-Sectional Study

**DOI:** 10.2196/49253

**Published:** 2024-01-09

**Authors:** Mingli Pang, Jieru Wang, Mingyue Zhao, Rui Chen, Hui Liu, Xixing Xu, Shixue Li, Fanlei Kong

**Affiliations:** 1 Centre for Health Management and Policy Research School of Public Health, Cheeloo College of Medicine Shandong University Jinan China; 2 NHC Key Lab of Health Economics and Policy Research Shandong University Jinan China; 3 Institute of Health and Elderly Care Shandong University Jinan China; 4 Human Resource Department The Second Hospital of Shandong University Jinan China

**Keywords:** loneliness, social support, sleep disturbance, older adults, migrant-local difference, structural equation modeling

## Abstract

**Background:**

Driven by the accelerated aging of the population of China, the number of older adults has increased rapidly in the country. Meanwhile, following children, migrant older adults (MOA) have emerged as a vulnerable group in the process of fast urbanization. Existed studies have illustrated the association between social support and loneliness and the relationship between sleep disturbance and loneliness; however, the underlying mechanisms and the migrant-local difference in the association between social support, sleep disturbance, and loneliness have not been identified.

**Objective:**

This study aimed to clarify the migrant-local difference in the relationship between social support, sleep disturbance, and loneliness in older adults in China.

**Methods:**

Multistage cluster random sampling was used to select participants: 1205 older adults (n=613, 50.9%, MOA and n=592, 49.1%, local older adults [LOA]) were selected in Weifang City, China, in August 2021. Loneliness was assessed with the 6-item short-form University of California, Los Angeles Loneliness Scale, social support was evaluated with the Social Support Rating Scale, and sleep disturbance was measured with the Pittsburgh Sleep Quality Index. The chi-square test, t test, and structural equation modeling (SEM) were adopted to explore the migrant-local difference between social support, sleep disturbance, and loneliness among the MOA and LOA.

**Results:**

The mean score of loneliness was 8.58 (SD 3.03) for the MOA and 8.00 (SD 2.79) for the LOA. SEM analysis showed that social support exerts a direct negative effect on both sleep disturbance (standardized coefficient=–0.24 in the MOA and –0.20 in the LOA) and loneliness (standardized coefficient=–0.44 in the MOA and –0.40 in the LOA), while sleep disturbance generates a direct positive effect on loneliness (standardized coefficient=0.13 in the MOA and 0.22 in the LOA).

**Conclusions:**

Both MOA and LOA have a low level of loneliness, but the MOA show higher loneliness than the LOA. There is a negative correlation between social support and loneliness as well as between social support and sleep disturbance among the MOA and LOA (MOA>LOA), while loneliness is positively associated with sleep disturbance in both populations (MOA<LOA). Measures should be taken by the government, society, and families to increase social support, decrease sleep disturbance, and further reduce the loneliness among older adults, especially the MOA.

## Introduction

Loneliness is typically defined as the discrepancy between a person’s desired and actual social relationships [1], which would increase the risk of other mental health problems [2,3] and physical health [4]. All people face loneliness at some stage in life [5]. Loneliness varies at different ages, and those aged less than 25 years and those aged over 65 years demonstrate the highest levels of loneliness [6]; data shows that up to one-third of older adults feel lonely [7].

Migration is found to be a risk factor for loneliness [8]. A study among ethnic-migrant groups in Australia showed that migrants from non-English-speaking countries demonstrate higher levels of loneliness than native-born, nonindigenous Australians [8]. Meanwhile, a study in Canada also illustrated that immigrants report higher levels of loneliness than native-born Canadians [9]. Many studies have shown that compared with local older adults (LOA), migrant older adults (MOA) tend to report higher loneliness [10,11]. These findings make it worthwhile to focus on the migrant-local difference between older adults’ loneliness.

Social support is an important determinant of loneliness [12,13]. A study among older adults in South Korea showed that social support is negatively associated with loneliness [14]. Moreover, research among older persons in the United Kingdom revealed that lower social support during the pandemic increased the risk of experiencing loneliness [15]. Meanwhile, a study among women during the postpartum period also showed that poorer social support would generally indicate a higher potential risk of loneliness [16].

A previous study showed that poor sleep is an independent risk factor for poor mental health in older adults [17]. A previous study in Denmark among young adolescents reported that loneliness is correlated with poor sleep problems [18]. One research among the rural older adults of China found that poor sleep quality is associated with increased odds of loneliness [19]. A study among US adults illustrated that more severe insomnia symptoms are significantly related to more feelings of loneliness [20]. Meanwhile, many studies have shown that a disrupted circadian rhythm/sleep loss and sleep problems could cause loneliness [21-23]. These findings indicate that sleep is linked to loneliness.

Existing studies have shown that people with poor social support [24] have poor sleep quality [25], and social support is found to be significantly associated with sleep disturbance [26]. Those with instrumental support can reduce the risk of all sleep problems, and having emotional support can reduce the risk of poor sleep quality [27]. A previous study among college students in China also found that perceived social support is significantly and negatively associated with insomnia [28].

In summary, the relationship between social support and loneliness, between social support and sleep disturbance, and between sleep disturbance and loneliness has been separately explored in previous studies. However, few studies have investigated the association between social support, sleep disturbance, and loneliness simultaneously related to the migrant-local difference. Thus, this study aimed to identify the migrant-local difference in the relationship between social support, sleep disturbance, and loneliness among older adults in China.

## Methods

### Participants

A total of 1205 older adults were selected in Weifang City, China, in August 2021: 613 (50.9%) MOA and 592 (49.1%) LOA. Up to November 30, 2020, the total household population of Weifang City was 9.39 million, of which 2.04 million, accounting for 21.7% of the total population, were aged 60 years and above, and the migrant population in the city was 2.38 million [29].

Multistage cluster random sampling was used to select participants. In the first stage, 4 districts were selected as primary sampling units (PSUs) based on the economic development and geographical location of Weifang City. In the second stage, in total, 4 subdistricts from the PSUs were selected as secondary sampling units (SSUs). In the third stage, in total, 4 communities were selected from the SSUs. The population aged 60 years or above in the selected communities in Weifang City constituted the entire sample. The inclusion criteria for participants were (1) age 60 years and above and (2) clear awareness and cognition. The MOA’s household registration was beyond Weifang City, while the LOA’s household registration was in Weifang City.

After completing training on the research background, questionnaire content, and social survey techniques, a total of 25 university students were assigned as interviewers for this study. Approximately 20-minute face-to-face interviews were conducted with each participant. Initially, 1208 older adults were selected and interviewed. However, 3 (0.2%) participants were excluded as they answered their questionnaires incorrectly or incompletely. Ultimately, a total of 1205 (99.8%) participants were included in the sample.

### Assessment and Measurements

#### Sociodemographic Characteristics

Sociodemographic characteristics included the following: gender (male, female), age (60-65, 66-70, 71-80, >80 years), marital status (married, single/divorced/separated/widowed), education level (primary school and below, junior high school, high school and above), job before retirement (agriculture, forestry, animal husbandry and fishery, others), and monthly household income (first quartile [Q1], second quartile [Q2], third quartile [Q3], fourth quartile [Q4]; Q1 was the poorest, and Q4 was the richest).

#### Loneliness

Loneliness is measured with the University of California at Los Angels (UCLA) Loneliness Scale. This scale includes 20 items, which range from 1 (never) to 4 (often) [30]. In this study, we used the 6-item short-form UCLA Loneliness Scale (ULS-6) to assess loneliness among older adults. The ULS-6 includes 6 items, with higher scores indicating higher loneliness [31]. The ULS-6 is suitable to use among older adults, and its reliability and validity in Chinese individuals has been verified [32,33], with a Cronbach α coefficient of .82 in this study.

#### Social Support

The Social Support Rating Scale (SSRS), developed by Xiao [34], was used to measure social support. The SSRS includes 10 items and 3 dimensions: objective social support, subjective social support, and social support use. The higher the score, the higher the social support level. The SSRS is appropriate to use among older adults [35], and the Cronbach α coefficient was .822 in this study.

#### Sleep Disturbance

The Pittsburgh Sleep Quality Index (PSQI) was used to assess the sleep disturbance of the participants in this study [36]. The PSQI consists 19 self-reported items divided into 7 components. The total score ranges from 0 to 21, and higher scores indicate severer sleep disturbance. The PSQI is appropriate to use among older adults [37,38], and its reliability and validity in Chinese individuals has been verified [39,40], with a Cronbach α coefficient of .731 in this study.

### Statistical Analysis

Descriptive statistics was used to clarify the sociodemographic characteristics of the older adults. The migrant-local difference between the sociodemographic characteristics among older adults was investigated using the chi-square test, and the migrant-local difference between social support, sleep disturbance, and loneliness was explored using the *t* test. Statistical significance was defined as *P*<.05. All statistical analyses were performed using SPSS version 24.0 (SPSS Inc). Structural equation modeling (SEM) was performed to investigate the impact of the migrant-local difference on the relationship between social support, sleep disturbance, and loneliness among older adults in Weifang City. The best-fit model was estimated using maximum likelihood estimation. The model fitness indices goodness-of-fit index (GFI), adjusted goodness-of-fit index (AGFI), comparative fit index (CFI), and root mean square error of approximation (RMSEA) were used; hypothetical models are considered well fitted to the data when GFI>0.90, AGFI>0.90, CFI>0.90, and RMSEA<0.05 [41]. SEM was conducted using AMOS 24.0 (IBM Corp).

### Ethical Considerations

The survey and data were obtained with written informed consent from all participants. The research program of the study was reviewed and approved by the Institutional Review Board (IRB) of Public Health and Preventive Medicine in Shandong University (#20180225) and was in accordance with the principles of the 1964 Declaration of Helsinki and its later amendments or comparable ethical standards.

## Results

### Characteristics of the Participants

Table 1 demonstrates the sociodemographic characteristics of the participants. We included 1205 older adults in the analysis, of which 613 (50.9%) were MOA and 592 (49.1%) were LOA. The MOA were mostly female (n=448, 73.1%); more than half (n=342, 55.8%) were 60-65 years old; most of them were married (n=539, 87.9%); 346 (56.4%) had primary school education or less; 461 (75.2%) were occupied in agricultural, forestry, and animal husbandry and fishery industries; 217 (35.4%) belonged to the Q1 (the poorest quartile) monthly household income group; 351 (57.3%) had no chronic disease; 335 (54.6%) had physical pain; and 195 (31.8%) reported a good health status. The LOA were also mostly women (n=437, 73.8%); 192 (32.4%) were 60-65 years old; 432 (73%) were married; 287 (48.5%) had primary school education or less; 400 (67.6%) worked in occupations other than agricultural, forestry, and animal husbandry and fishery industries; 219 (37%) belonged to the Q4 (the richest quartile) monthly household income group; 355 (60%) had chronic disease; 351 (59.3%) had physical pain; and 169 (28.5%) reported a good health status. The differences between the MOA and the LOA were statistically significant for age (*P*<.001), marital status (*P*<.001), education level (*P*<.02), job before retirement (*P*<.001), monthly household income (*P*<.001), chronic disease (*P*<.001), and self-reported health status (*P*<.001).

Table 2 shows the characteristics of social support, sleep quality, and loneliness among the MOA and LOA. The mean total score of loneliness was 8.58 (SD 3.03) for the MOA and 8.00 (SD 2.79) for the LOA. Specifically, the mean scores of individual items were as follows: often feel a lack of friends, 1.53 (SD 0.79) for the MOA and 1.36 (SD 0.71) for the LOA; often feel no one can be trusted, 1.47 (SD 0.73) for the MOA and 1.37 (SD 0.71) for the LOA; often feel left out, 1.37 (SD 0.62) for the MOA and 1.28 (SD 0.55) for the LOA; often feel separated from others, 1.37 (SD 0.67) for the MOA and 1.30 (SD 0.64) for the LOA; often feel shy, 1.36 (SD 0.64) for the MOA and 1.25 (SD 0.52) for the LOA; and often feel surrounded by people but not cared for, 1.49 (SD 0.72) for the MOA and 1.43 (SD 0.71) for the LOA. Statistically significant differences were found for the total score (*t*_1203_=–3.442, *P*=.01), often feel a lack of friends (*t*_1203_=–3.704, *P*<.001), often feel no one can be trusted (*t*_1203_=–2.326, *P*=.02), often feel left out (*t*_1203_=–2.672, *P*=.008), and often feel shy (*t*_1203_=–3.365, *P*=.001) between the MOA and the LOA.

**Table 1:** Sociodemographic characteristics of the MOA^a^ and LOA^b^ by migrant-local difference in Weifang City, China, in August 2021.

Characteristics		Total (N=1205), n (%)	Migrant-local difference	
			MOA (n=613), n (%)	LOA (n=592), n (%)
**Sex; *χ*^2^_1_=0.083, *P*=.77**				
	Male	320 (26.6)	165 (26.9)	155 (26.2)
	Female	885 (73.4)	448 (73.1)	437 (73.8)
**Age (years); *χ*^2^_3_=131.429, *P*<.001**				
	60-65	534 (44.3)	342 (55.8)	192 (32.4)
	66-70	301 (25.0)	171 (27.9)	130 (22.0)
	71-80	256 (21.2)	80 (13.1)	176 (29.7)
	>80	114 (9.5)	20 (3.2)	94 (15.9)
**Marital status; *χ*^2^_1_=43.045, *P*<.001**				
	Married	971 (80.6)	539 (87.9)	432 (73.0)
	Single/divorced/separated/widowed	234 (19.4)	74 (12.1)	160 (27.0)
**Education level; *χ*^2^_2_=7.662, *P*=.02**				
	Primary school and below	633 (52.5)	346 (56.4)	287 (48.5)
	Junior high school	338 (28.0)	158 (25.8)	180 (30.4)
	High school and above	234 (19.5)	109 (17.8)	125 (21.1)
**Job before retirement; *χ*^2^_1_=221.935, *P*<.001**				
	Agricultural, forestry, animal husbandry and fishery	653 (54.2)	461 (75.2)	192 (32.4)
	Others	552 (45.8)	152 (24.8)	400 (67.6)
**Monthly household income; *χ*^2^_3_=158.680, *P*<.001**				
	Q1^c^	301 (25.0)	217 (35.4)	84 (14.2)
	Q2^d^	301 (25.0)	194 (31.6)	107 (18.1)
	Q3^e^	302 (25.0)	120 (19.6)	182 (30.7)
	Q4^f^	301 (25.0)	82 (13.4)	219 (37.0)
**Chronic disease; *χ*^2^_1_=35.765, *P*<.001**				
	Yes	617 (51.2)	262 (42.7)	355 (60.0)
	No	588 (48.8)	351 (57.3)	237 (40.0)
**Physical pain; *χ*^2^_1_=2.646, *P*=.104**				
	Yes	686 (56.9)	335 (54.6)	351 (59.3)
	No	519 (43.1)	278 (45.4)	241 (40.7)
**Self-reported health status; *χ*^2^_5_=35.289, *P*<.001**				
	Bad	89 (7.4)	30 (4.9)	59 (10.0)
	Relatively bad	29 (2.4)	15 (2.4)	14 (2.4)
	Average	251 (20.8)	113 (18.4)	138 (23.2)
	Relatively good	233 (19.3)	106 (17.3)	127 (21.5)
	Good	364 (30.3)	195 (31.8)	169 (28.5)
	Very good	239 (19.8)	154 (25.2)	85 (14.4)

^a^MOA: migrant older adults.

^b^LOA: local older adults.

^c^Q1: first quartile (poorest).

^d^Q2: second quartile

^e^Q3: third quartile.

^f^Q4: fourth quartile (richest).

**Table 2:** General characteristics of social support, sleep disturbance, and loneliness among the MOA^a^ and LOA^b^ by the migrant-local difference using the *t* test in Weifang City, China, in August 2021.

Variables			Total (N=1205), mean (SD)		Migrant-local difference				*t*_1203_ Test		*P* value
					MOA (n=613), mean (SD)		LOA (n=592), mean (SD)				
**Social support (SSRS^c^)**											
	Total	38.94 (6.61)		38.89 (6.63)		39.51 (6.86)		1.612		.11	
	Objective social support	8.22 (2.01)		8.47 (1.64)		7.95 (2.30)		–4.545		<.001	
	Subjective social support	23.68 (4.47)		23.47 (4.79)		24.43 (4.40)		3.608		<.001	
	Social support use	7.04 (2.35)		6.94 (2.26)		7.14 (2.44)		1.432		.15	
**Sleep disturbance (PSQI^d^)**											
	Total	4.44 (3.66)		4.29 (3.57)		4.58 (3.66)		1.396		.16	
	Subjective sleep quality	0.92 (0.84)		0.88 (0.83)		0.97 (0.86)		1.968		.05	
	Sleep latency	1.12 (1.20)		1.11 (1.21)		1.12 (1.19)		0.108		.91	
	Sleep continuity	0.61 (0.91)		0.54 (0.84)		0.68 (0.97)		2.822		.005	
	Habitual sleep efficiency	0.17 (0.53)		0.16 (0.50)		0.18 (0.56)		0.624		.53	
	Sleep disorder	0.97 (0.57)		0.97 (0.56)		0.97 (0.57)		0.170		.87	
	Use of sleep medicine	0.15 (0.59)		0.13 (0.55)		0.18 (0.62)		1.482		.14	
	Daytime dysfunction	0.50 (0.77)		0.52 (0.78)		0.48 (0.77)		–0.765		.45	
**Loneliness(ULS-6^e^)**											
	Total	8.29 (2.93)		8.58 (3.03)		8.00 (2.79)		–3.442		.001	
	Often feel lack of friends	1.45 (0.76)		1.53 (0.79)		1.36 (0.71)		–3.704		<.001	
	Often feel no one can be trusted	1.42 (0.72)		1.47 (0.73)		1.37 (0.71)		–2.326		.02	
	Often feel left out	1.32 (0.59)		1.37 (0.62)		1.28 (0.55)		–2.672		.008	
	Often feel separated from others	1.34 (0.65)		1.37 (0.67)		1.30 (0.64)		–1.76		.08	
	Often feel shy	1.31 (0.58)		1.36 (0.64)		1.25 (0.52)		–3.365		.001	
	Often feel surrounded by people but not cared for	1.46 (0.71)		1.49 (0.72)		1.43 (0.71)		–1.265		.21	

^a^MOA: migrant older adults.

^b^LOA: local older adults.

^c^SSRS: Social Support Rating Scale.

^d^PSQI: Pittsburgh Sleep Quality Index.

^e^ULS-6: 6-item short-form University of California, Los Angeles (UCLA) Loneliness Scale.

The mean total score of sleep disturbance was 4.29 (SD 3.57) for the MOA and 4.58 (SD 3.66) for the LOA. Specifically, the mean scores of individual items were as follows: subjective sleep quality, 0.88 (SD 0.83) for the MOA and 0.97 (SD 0.86) for the LOA; sleep latency, 1.11 (SD 1.21) for the MOA and 1.12 (SD 1.19) for the LOA; sleep continuity, 0.54 (SD 0.84) for the MOA and 0.68 (SD 0.97) for the LOA; habitual sleep efficiency, 0.16 (SD 0.50) for the MOA and 0.18 (SD 0.56) for the LOA; sleep disorder, 0.97 (SD 0.56) for the MOA and 0.97 (SD 0.57) for the LOA; use of sleep medicine, 0.13 (SD 0.55) for the MOA and 0.18 (SD 0.62) for the LOA; and daytime dysfunction, 0.52 (SD 0.78) for the MOA and 0.48 (SD 0.77) for the LOA. Statistically significant differences were found for subjective sleep quality (*t*_1203_=1.968, *P*=.05) and sleep continuity (*t*_1203_=2.822, *P*=.005) between the MOA and the LOA.

The mean total score of social support was 38.89 (SD 6.63) for the MOA and 39.51 (SD 6.86) for the LOA. Specifically, the mean scores of individual items were as follows: objective social support, 8.47 (SD 1.64) for the MOA and 7.95 (SD 2.30) for the LOA; subjective social support, 23.47 (SD 4.79) for the MOA and 24.43 (SD 4.40) for the LOA; and social support use, 6.94 (SD 2.26) for the MOA and 7.14 (SD 2.44) for the LOA. Statistical differences were found for objective social support (*t*_1203_=–4.545, *P*<.001) and subjective social support (*t*_1203_=3.608, *P*<.001) between the MOA and the LOA.

### The Structural Model

#### Measurement Invariance Across the Migrant and Local Groups

Before the discussion of the migrant-local difference in the structural model, multigroup model invariance should be first tested. Partial measurement invariance was used to clarify the measurement invariance about the multigroup models in this study, which could assess the invariance with ∆CFI (change in the CFI) and ∆RMSEA (change in the RMSEA) for comparing the less restricted model with the more constrained model. The basic test strategy was to outline correspondence to model trimming, where an initial unconstrained model was gradually restricted by adding constrains [42]. Table 3 illustrates the related fit statistics of the measures' invariance across the migrant-local difference and the fitness index for the 7 chosen models. Fitness indices of the MOA (M1) and LOA (M2) models were first compared to test whether the variable migrant-local difference was suitable for group comparison. As shown in Table 3, the model fitness indices between the M1 and M2 were ∆CFI=0 and ∆RMSEA=0, indicating that the different effects between the MOA and LOA could be compared.

Next, the measurement invariance was evaluated with ∆CFI and ∆RMSEA between the unconstrained model (M3), the measurement weights model (M4), the structural weights model (M5), the structural covariances model (M6), and the structural residuals model (M7). M3 did not restrict any coefficients, M4 supposed that the indicator loadings for the corresponding construct in each group were the same, M5 constrained both indicator loadings for the corresponding construct and the structural coefficients across the groups, M6 assumed that the indicator loadings for the corresponding construct and the structural coefficients across the groups were equal and also that the covariance of the endogenous variables across the groups were equal, and M7 assumed that the indicator loadings, structural coefficients, covariance of the endogenous variables, and variance of the exogenous variable were equal across the groups.

**Table 3:** Multigroup model invariance test results using AMOS software. Variables in 7 models were social support, sleep disturbance, and loneliness among the MOA^a^ and LOA^b^ in Weifang City, China, in August 2021 (N=1205).

Model	*χ*^2^ (*df*)	*P* value	*χ*^2^/*df*	GFI^c^	AGFI^d^	CFI^e^	RMSEA^f^	∆CFI^g^	∆RMSEA^h^
M1^i^	579.952 (202)	<.001	2.871	0.941	0.920	0.925	0.039	—^j^	—
M2^k^	579.952 (202)	<.001	2.871	0.941	0.920	0.925	0.039	0	0
M3^l^	579.952 (202)	<.001	2.871	0.941	0.920	0.925	0.039	0	0
M4^m^	593.973 (215)	<.001	2.763	0.939	0.923	0.925	0.038	0	–0.001
M5^n^	597.464 (218)	<.001	2.741	0.939	0.924	0.924	0.038	–0.001	–0.001
M6^o^	598.632 (219)	<.001	2.733	0.939	0.924	0.924	0.038	–0.001	–0.001
M7^p^	601.83 (221)	<.001	2.723	0.939	0.924	0.924	0.038	–0.001	–0.001

^a^MOA: migrant older adults.

^b^LOA: local older adults.

^c^GFI: goodness-of-fit index.

^d^AGFI: adjusted goodness-of-fit index.

^e^CFI: comparative fitness index.

^f^RMSEA: root mean square error of approximation.

^g^∆CFI: change in the CFI.

^h^∆RMSEA: change in the RMSEA.

^i^M1: MOA model.

^j^Not applicable.

^k^M2: LOA model.

^l^M3: unconstrained model.

^m^M4: measurement weights model.

^n^M5: structural weights model.

^o^M6: structural covariances model.

^p^M7: structural residuals model.

According to Table 3, ∆CFI was 0 between M4 and M3 and –0.001 between M5 and M4, between M6 and M5, and between M7 and M6, while ∆RMSEA was –0.001 between M4 and M3, between M5 and M4, between M6 and M5, and between M7 and M6. The fact that all the ∆CFI values were <0.010 and all the ∆ RMSEA values were <0.015 indicated that the M1, M2, M3, M4, M5, M6, and M7 models had established measurement invariance across the migrant-local difference [43]. The comparison of the values between different variables across the MOA and LOA was thus established.

#### Model Fitness Indices

**Figure 1:**
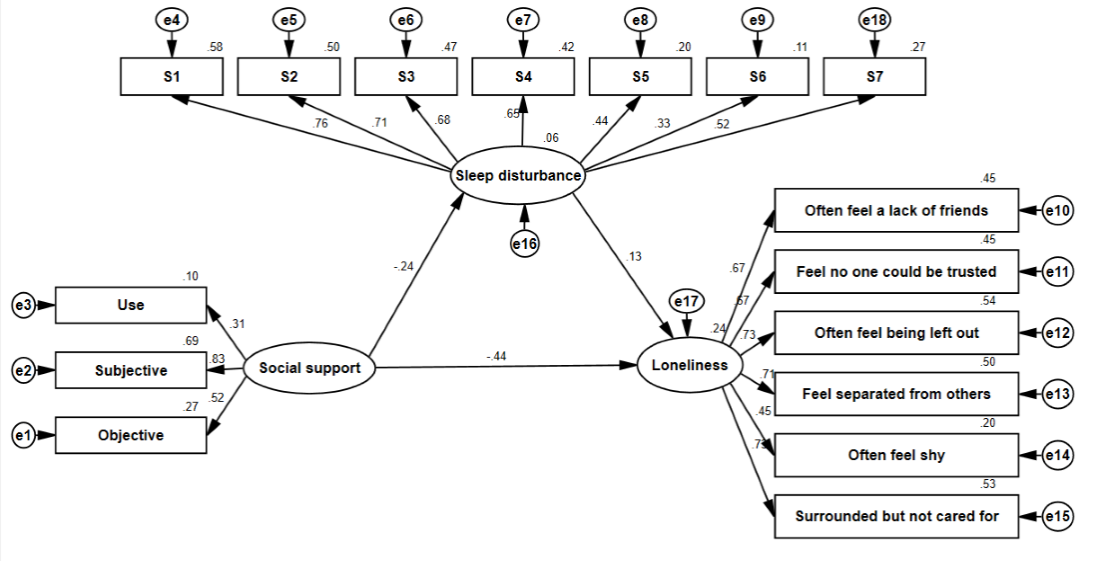
SEM analysis of the association between social support, sleep disturbance, and loneliness of MOA (n=613) in Weifang City, China, in August 2021. e: residual variables; MOA: migrant older adults; S1: subjective sleep quality; S2: sleep latency; S3: sleep continuity; S4: habitual sleep efficiency; S5: sleep disorder; S6: use of sleep medicine; S7: daytime dysfunction; SEM: structural equation modeling.

**Figure 2:**
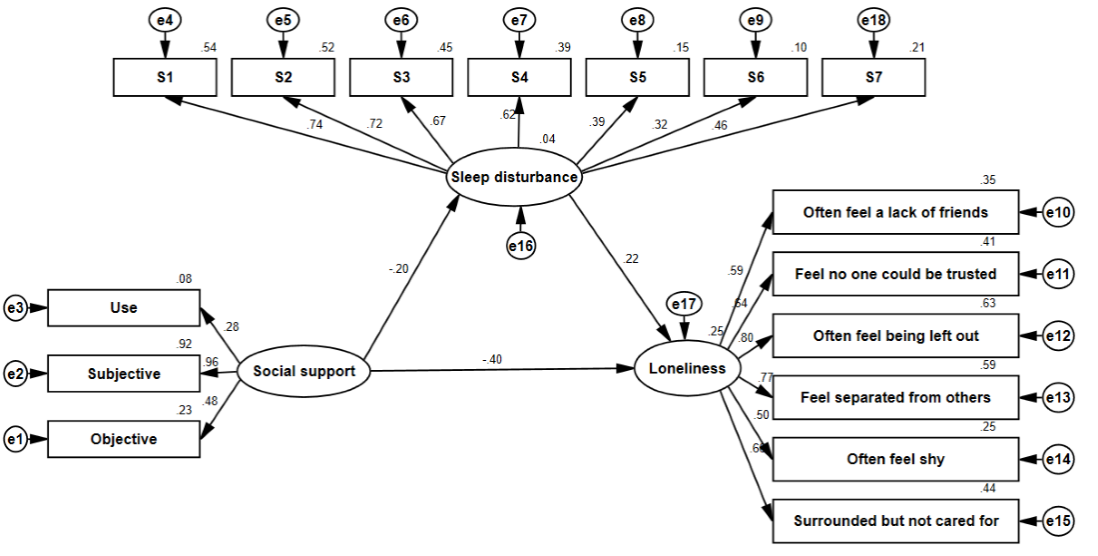
SEM analysis of the association between social support, sleep disturbance, and loneliness of LOA (n=592) in Weifang City, China, in August 2021. e: residual variables; LOA: local older adults; S1: subjective sleep quality; S2: sleep latency; S3: sleep continuity; S4: habitual sleep efficiency; S5: sleep disorder; S6: use of sleep medicine; S7: daytime dysfunction; SEM: structural equation modeling.

The proposed unconstrained model (M3) for the MOA and LOA is illustrated in Figures 1 and 2, respectively, and 3 latent variables (social support, sleep disturbance, and loneliness) were included in the model; the model fitness indices are also presented in Table 3. The estimates of model fitness were similar for both groups: GFI=0.941>0.90, AGFI=0.920>0.90, CFI=0.925>0.90, and RMSEA=0.039<0.05, implying that the proposed model well fitted the empirical data for both the MOA and the LOA in this study.

### Relationship Between Social Support, Sleep Disturbance, and Loneliness

The relationship between social support, sleep disturbance, and loneliness is illustrated in Table 4 and in Figures 1 and 2 among the MOA and LOA, respectively.

**Table 4:** Standardized effects between social support, sleep disturbance, and loneliness among the MOA^a^ and LOA^b^ by the migrant-local difference in Weifang, China, in August 2021 (N=1205).

Variable	Direct effects			Indirect effects	
	MOA	LOA	MOA		LOA
Social support → loneliness	–0.44 (*P*<.001)	–0.40 (*P*<.001)	—^c^		—
Sleep disturbance → loneliness	0.13 (*P*=.03)	0.22 (*P*<.001)	—		—
Social support → sleep disturbance	–0.24 (*P*<.001)	–0.20 (*P*<.001)	—		—
Social support → sleep disturbance → loneliness	—	—	–0.03 (*P*<.001)		–0.04 (*P*<.001)

^a^MOA: migrant old adults.

^b^LOA: local old adults.

^c^Not applicable.

#### Association Between Social Support and Loneliness

Social support and its indicators exerted a negative effect on loneliness. The direct negative effect of social support on loneliness was found among both MOA (standardized coefficient=−0.44) and LOA (standardized coefficient=−0.40), although the effect was stronger in the MOA than in the LOA. Additionally, the standardized effect of social support on loneliness was statistically significant in both groups. The results indicated that higher social support would result in less loneliness among both MOA and LOA.

#### Association Between Sleep Disturbance and Loneliness

Sleep disturbance exerted a direct positive effect on loneliness among both MOA (standardized coefficient=0.13) and LOA (standardized coefficient=0.22), although the effect was stronger in the LOA than in the MOA. Additionally, the standardized effect of sleep disturbance on loneliness was statistically significant in both groups. The results implied severer that sleep disturbance among both MOA and LOA would result in higher loneliness.

#### Association Between Social Support and Sleep Disturbance

Social support had a direct negative effect on sleep disturbance among both MOA (standardized coefficient=–0.24) and LOA (standardized coefficient=–0.20), although the effect was stronger in the LOA than in the MOA. A statistically significant relationship between social support and sleep quality was found in both groups. The results indicated that higher social support would result in less sleep disturbance.

## Discussion

### Principal Findings

The mean score of loneliness among the MOA (mean 8.58, SD 3.03) and the LOA (mean 8.00, SD 2.79) indicated a fairly low level of loneliness in both groups in this research, also lower than the mean score of loneliness reported in a previous study among the rural empty-nest older adults in China (mean 16.19, SD 3.90) [44]. Moreover, the MOA showed a mildly higher loneliness than the LOA in this study, which is similar to a previous study that showed that the mean score of loneliness is higher among older Turkish German adults than their native-born German counterparts [10].

Using SEM, this study investigated the migrant-local difference in the association between social support, sleep disturbance, and loneliness among MOA and LOA. First, the results illustrated that social support affected loneliness among both MOA and LOA, although the effect was stronger in the MOA than in the LOA. Second, sleep disturbance exerted a direct positive effect on loneliness among both MOA and LOA, although the effect was stronger in the LOA than in the MOA. Third, social support affected sleep disturbance negatively and directly among both MOA and LOA, although the effect was stronger in the MOA than in the LOA.

### Relationship Between Social Support and Loneliness

A negative relationship between social support and loneliness was found among both MOA and LOA, which is consistent with previous research [45,46]. The association between social support and loneliness can be explained by the main effect and buffer effect models [47-49]. The main effect model holds that social support has a universal gain effect and that any increase in social support is bound to result in an increase in health status (eg, a lower level of loneliness), regardless of an individual’s social support level. In the buffer effect model, social support is only associated with physical and mental health in stressful situations, which cushions the negative effects of stressful events on physical and mental health and maintains or improves an individual’s physical and mental health [50]. Through these 2 models, social support can increase positive emotional experiences, control negative emotional experiences, and finally reduce loneliness.

### Relationship Between Sleep Disturbance and Loneliness

Sleep disturbance was found could exert a positive effect on loneliness among both MOA and LOA, which is similar to a previous study that showed that individuals with poor sleep quality report higher levels of loneliness [51]. A study among adolescents also showed that loneliness is significantly positively associated with sleep disturbance [52]. The positive association between sleep disturbance and loneliness may be due to the result of sleep disturbance–related metabolic, neural, and hormonal processes [51]; moreover, sleep disturbance may result in exhaustion in older adults, which further reduces their communication with others, leading to loneliness.

### Relationship Between Social Support and Sleep Disturbance

A negative association between social support and sleep disturbance was found among both MOA and LOA, which is similar to previous studies that have reported that higher social support would generally predict less sleep disturbance [53-55]. Previous studies have also shown that sleep disturbance is significantly associated with social support among Chinese medical staff [56], as well as young Chinese rural residents [57] in China. Moreover, a meta-analysis showed a robust association between social support and favorable sleep outcomes [58]; that is, higher social support would result in less sleep disruption and less sleep disturbance.

### Migrant-Local Difference in the Relationship Between Social Support, Sleep Disturbance, and Loneliness Among MOA and LOA

Regarding the migrant-local difference, a negative effect of social support on loneliness was found among both MOA and LOA, although the effect was higher in the MOA than in the LOA. The difference between these 2 groups may be because the MOA migrated to a new place and faced many issues with adaptation, such as acculturation stress [59,60], a low sense of psychological identity [61], and less use of outpatient mental health care services [62]; all these factors would increase their loneliness. In addition, after moving to the city where their children lived, the support network of the MOA in their hometowns broke but a new urban support network was not yet formed; consequently, the MOA faced a situation in which they lacked social support [63]. Thus, compared with the LOA, the MOA were more lonely with less social support, so the effect of social support on loneliness was higher among the MOA than the LOA.

Regarding the migrant-local difference, a direct positive effect of sleep disturbance on loneliness was found among both MOA and LOA, although the effect was higher in the LOA than in the MOA. This difference between the 2 groups may be because the LOA in this study were older and more of them were single/divorced/separated/widowed compared to the MOA, and these 2 factors (age and marital status) have been found to be negatively associated with sleep quality in previous studies [64,65]; thus, compared with MOA, LOA would be likely to have more sleep disturbance. A previous study illustrated that insomnia is not only a symptom of mood disorders but also a risk factor for mental disorder development [66]. Therefore, compared with MOA, LOA have more severe sleep disturbance; furthermore, severe sleep disturbance exerts more effect on loneliness among LOA.

Considering the migrant-local difference, an association was found between social support and sleep disturbance among both MOA and LOA, but it was slightly stronger among the MOA than the LOA. This may be because compared with the LOA, the MOA faced reduced social support [67], more social alienation [68], and worse social integration [69]. Among the MOA, after migration to a new city, because of support network reconstruction and conservative awareness, it is difficult to adapt to the local community in a short time [70]. All these factors make MOA more dependent on social support, and further, social support has a stronger effect on sleep disturbance among MOA compared with LOA.

### Implications

Due to the migrant-local difference, different supportive measures need to be targeted for MOA and LOA. First, since the negative relationship between social support and loneliness in this study was stronger in the MOA, family members should provide more social support for older adults, especially for MOA, and provide them with more knowledge about the importance and prevention of loneliness, while providing social support. Second, the community should design more programs and create activities for MOA, which could increase the interpersonal communication between the MOA, as well as between the MOA and the LOA, by enhancing their peer social support, thus decreasing sleep disturbance and loneliness among both groups, especially the MOA. Third, since the positive relationship between sleep disturbance and loneliness in this study was stronger among the LOA, it is suggested that the community pay more attention to LOA (eg, no loud noises at night), while the family members should create a better sleeping environment (eg, adequate sleeping temperature and well-ventilated bedroom). Fourth, community health care providers should provide more mental health monitoring for both MOA and LOA, especially for older adults with sleep disturbance. Fifth, the findings of this study could attract more Chinese scholars to focus on the level of loneliness among MOA and LOA, especially sociocultural and physical health status contributors, and conduct comparative research between different areas among MOA and LOA in the future.

### Limitations

There are several limitations of this study. First, we used cross-sectional data, which could not be well explored for causal relationships. Second, socioeconomic status plays a fundamental role in the study of population health, which was not explored in this study; we will examine this more in future studies. Third, some other factors that may also affect loneliness among MOA and LOA (eg, stress and depression) were not included in the study, which will be considered in future studies. Fourth, many other factors (eg, medication usage) might influence the sleep quality in older adults, and information related to health status is also a potential confounder of the effect of the quality of sleep, so future studies should examine whether other mediators can explain the internal mechanism underlying the relationship between sleep disturbance and social support.

### Conclusion

This study illustrated the migrant-local difference in the association between social support, sleep quality, and loneliness between MOA and LOA in Weifang City, China. The results showed that (1) social support exerts a negative effect on loneliness among both MOA and LOA, although the effect is higher in MOA; (2) loneliness is significantly and positively associated with sleep disturbance among both MOA and LOA, although the effect is higher in LOA; and (3) there is a negative association between social support and sleep disturbance among both MOA and LOA, yet it is slightly higher in the MOA. This study may provide helpful information on how to decrease and prevent loneliness among older adults by enhancing their social support and decreasing their sleep disturbance.

## Abbreviations

AGFI: adjusted goodness-of-fit index
CFI: comparative fit index
GFI: goodness-of-fit index
LOA: local older adults
MOA: migrant older adults
PSQI: Pittsburgh Sleep Quality Index
PSU: primary sampling unit
Q1: first quartile
Q2: second quartile
Q3: third quartile
Q4: fourth quartile
RMSEA: root mean square error of approximation
SEM: structural equation modeling
SSRS: Social Support Rating Scale
SSU: secondary sampling unit
UCLA: University of California at Los Angels
ULS-6: 6-item short-form University of California, Los Angeles (UCLA) Loneliness Scale

## Data Availability

The data sets generated and analyzed during this study are not publicly available due to privacy restrictions but are available from the corresponding author upon reasonable request.
